# Survival of skyrmions along granular racetracks at room temperature†

**DOI:** 10.1039/d3na00464c

**Published:** 2023-07-28

**Authors:** Josep Castell-Queralt, Guillermo Abad-López, Leonardo González-Gómez, Nuria Del-Valle, Carles Navau

**Affiliations:** a Departament de Física, Universitat Autònoma de Barcelona 08193 Bellaterra Barcelona Catalonia Spain carles.navau@uab.cat

## Abstract

Skyrmions can be envisioned as bits of information that can be transported along nanoracetracks. However, temperature, defects, and/or granularity can produce diffusion, pinning, and, in general, modification in their dynamics. These effects may cause undesired errors in information transport. We present simulations of a realistic system where both the (room) temperature and sample granularity are taken into account. Key feasibility magnitudes, such as the success probability of a skyrmion traveling a given distance along the racetrack, are calculated. The results are evaluated in terms of the eventual loss of skyrmions by pinning, destruction at the edges, or excessive delay due to granularity. The model proposed is based on the Fokker–Planck equation resulting from Thiele's rigid model for skyrmions. The results could serve to establish error detection criteria and, in general, to discern the dynamics of skyrmions in realistic situations.

## Abstract 2

The probability density of the survival of skyrmions when travelling along a racetrack is evaluated. The granularity of the track is considered by modeling the grains as local variations of the anisotropy. Room temperature is also considered.

## Graphical abstract

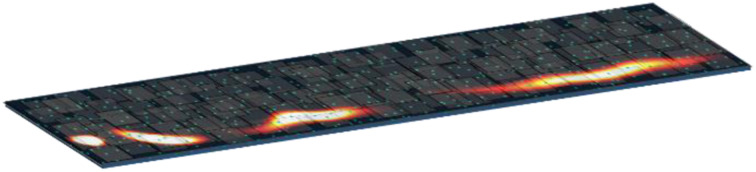

## 1 Introduction

Magnetic skyrmions are whirling magnetic structures that can be found in certain magnetic materials.^1^ Their small size and high mobility have promoted them as promising information carriers, as well as building blocks in ultradense magnetic memories, logic devices, and computational systems.^2–6^ In ferromagnetic ultrathin films, with a heavy-metal substrate, it has been found that skyrmions can be stabilized with the aid of the interfacial Dzyaloshinskii–Moriya interaction.^7–10^ The same mechanism allows the formation of skyrmions in multilayers with alternating ferromagnets (FMs) and heavy-metals.^11,12^ The experimental finding of room-temperature skyrmions^13^ has boosted the potentiality of skyrmions for applications and, consequently, the study of their spin-current driven dynamics at non-zero temperatures to predict the feasibility of the aforementioned applications.^14,15^

One of the most promising skyrmion-based applications proposed to date is the skyrmionic racetrack memory. It is designed to drive skyrmions along the racetrack, with the spin–orbit torque produced by a spin-polarized current fed into a heavy-metal substrate.^2,16,17^ In such systems, the borders of the track create a confining potential that sets a driving velocity upper threshold above which the skyrmions would escape the track.^18–21^ Hence, when transporting stable skyrmions at zero temperature along a clean (defect-free and grain-free) racetrack we have two possible scenarios depending on the driving current: either the skyrmion is annihilated at the edge or it is channeled along the racetrack.

However, real skyrmionic racetracks are granular, and one would like to operate at room temperature. It is known that granularity acts as an array of pinning potentials for skyrmions that results in a minimum applied current density for the activation of skyrmion motion.^22–30^ At room temperature, stochastic effects on the skyrmions' position^31–33^ could compromise their stability when approaching the borders or defects.^19,34^ Moreover, even above the minimum threshold, there is a certain probability of trapping the skyrmion.^35–37^

Consequently, in a real racetrack, there is no longer a binary scenario for the survival of skyrmions after a given length of track, for a given time. The problem becomes probabilistic. Here we address this problem by realistically simulating a nanoracetrack for skyrmion transport, taking into account both the (room) temperature and granularity. We use a deterministic, yet probabilistic, approach to study the dynamics of a skyrmion in a granular racetrack at room temperature. In the present work, we first model the interaction of the grains of the system with a skyrmion and derive the stochastic Thiele's equation (STE); second, we obtain the corresponding Fokker–Planck equation (FPE), which includes the temperature, and solve it numerically; and third, we study the feasibility of skyrmionic racetracks and how granularity affects the performance of such devices at room temperature.

## 2 General model

**Fig. 1:**
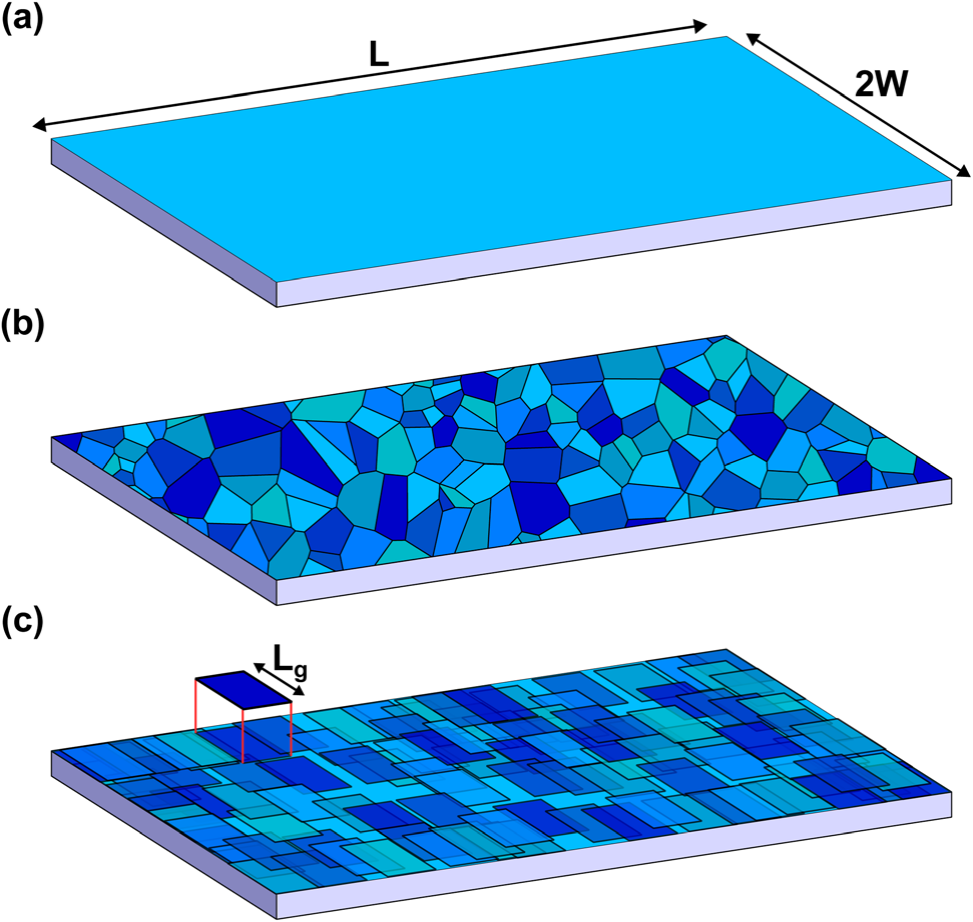
Sketch of the modeled racetrack. (a) Clean racetrack: the skyrmion is driven along the track a given length *L* and confined by the borders at *y* = ±*W*. (b) A Voronoi tessellation, showing a possible grain structure. (c) The grains are modeled as square regions randomly distributed in the plane with Gaussian-distributed anisotropy constants (represented by the different blue tones in the sketch).

The rigid (Thiele's) model^38^ assumes that, during motion, the internal structure of the skyrmion is not modified, so its position and velocity can be described by generalized magnitudes, **r**_s_ and **v**_s_ respectively. We consider that the skyrmion is moving along a thin ferromagnetic racetrack of length *L* and width 2*W*. The thickness of the ferromagnetic layer is *d* ≪ *W*, *L*. Thus, the magnetization is considered uniform across the thickness and we locate the FM layer at *z* = 0 (see the sketch in Fig. 1a). Considering symmetric Néel-type skyrmions on a background magnetization pointing to −**ẑ**, the STE for the movement of skyrmions driven by damping-like torques (produced by spin-polarized currents arising from the spin-Hall effect) after feeding an in-plane current **J**_H_ in a heavy-metal substrate is^39,40^1
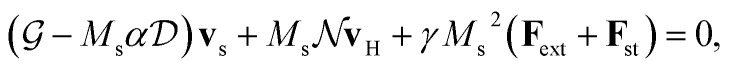
where 
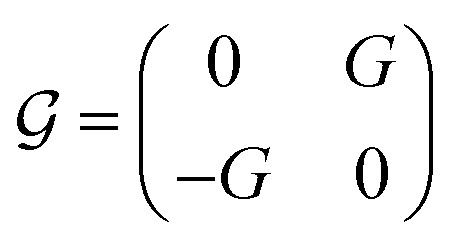
, 
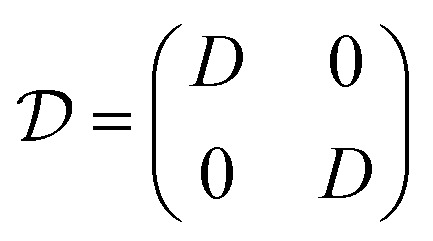
, and 
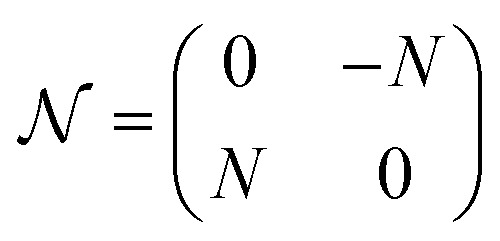
, with *G*, *D*, and *N* being constants that depend on the internal structure of the skyrmion. *α* is the Gilbert damping constant, *γ* (*γ* = 2.21 × 10^5^ m A^−1^ s^−1^) the gyromagnetic constant, 
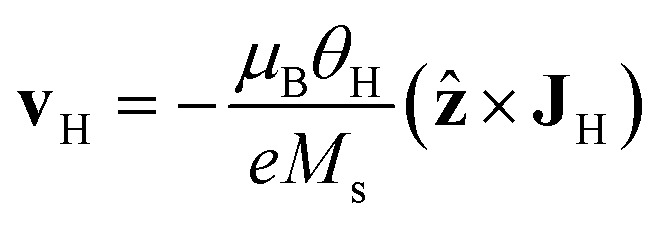
, with *μ*_B_ being the Bohr magneton, *θ*_H_ the Hall angle, *e* (>0) the charge of the electron, and *M*_s_ the saturation magnetization. We use *G* = 4π*dM*_s_^3^, *D* = 4π*dM*_s_^2^ and *N* = 4π*RM*_s_^2^, where *R* is the radius of the rigid skyrmion. These values correspond to ideal Néel skyrmions with *z*-magnetization *M*_*z*_ = *M*_s_(*R*^2^ − *ρ*^2^)/(*ρ*^2^ + *R*^2^). **F**_st_ and **F**_ext_ come from the stochastic and external forces, respectively. They will contain information about the temperature and granularity, as explained in the following subsections.

Note that we are assuming that the rigid model holds for the parameters used in this paper. As in all Thiele's approaches, this is valid as long as the skyrmions do not change considerably their internal structure. Thiele's approach has been used extensively to explain the movement of skyrmions when driving currents are moderate or when the skyrmions are far from annihilation (including defects and temperature) both theoretically and for understanding experimental measurements.^15,23,30,31,36,41–44^ The present model works in the regimes in which the skyrmions' deformation does not substantially affect their motion.

### 2.1 Temperature

The temperature *T* is introduced by considering the term **F**_st_ as a white noise with 〈*F*_st,*j*_〉 = 0 and 
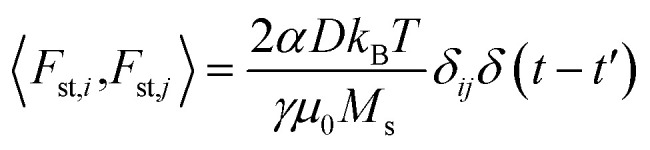
, where *i*, *j* = *x*, *y*, *z*. *μ*_0_ is the vacuum permeability, *k*_B_ the Boltzmann constant, *δ*_*ij*_ the Kronecker delta and *δ*(*t* − *t*′) the temporal Dirac's delta.^31^

### 2.2 Borders and granularity

The term **F**_ext_ in eqn (1) has two contributions, **F**_ext_ = **F**_R_ + **F**_G_. The force‡‡Strictly, **F**_ext_ and **F**_st_ do not have units of force. Nevertheless, we call them “force terms”, following the usual nomenclature. coming from the confining potential of the racetrack is **F**_R_ and the force produced by the granularity is **F**_G_.

#### 2.2.1 Borders

The edges of a racetrack create a confining potential that repels the skyrmion. However, if a large enough driving current is applied, this potential can be overcome and the skyrmion is annihilated when it reaches the edge. An exponential function decaying with the edge-skyrmion distance has been proposed to account for these facts.^18,45^ Thus, the force created by the borders of the racetrack over a skyrmion, whose generalized position is **r**_s_ = *x*_s_**x̂** + *y*_s_**ŷ**, is modeled as

2 **F**_R_ = *f*_0_[e^−(*y*_s_+*W*)/*R*^ − e^−(*y*_s_−*W*)/*R*^]**ŷ**,

where *f*_0_ is a parameter that controls the strength of the repulsion.

Since the rigid model does not allow us to consider the skyrmion deformation near the edges, we also assume that a skyrmion is annihilated when it “touches” any racetrack border, that is when the distance between its center and the edge equals the radius of the skyrmion.

#### 2.2.2 Granularity

To model the granularity force term, **F**_G_, is a more intricate task. The physical origin of this term arises from the different physical-property values of the different grains that form the FM films. Although all the relevant magnetic parameters can vary slightly between grains (*e.g.*, exchange constant, Dzyaloshinskii–Moriya constant, or *M*_s_) the most (usually) modified one is the uniaxial anisotropy constant, as the strength and the direction of the uniaxial anisotropy strongly depend on the crystallographic orientation of the material.^27,46^ For simplicity, we only consider variations of anisotropy in our model.

A common approach to model granularity within the micromagnetic model is to generate a random set of grains, using Voronoi tessellation,^36,47,48^ with an average size and some randomly distributed variation of anisotropy constant in each grain (see Fig. 1b). However, there is no clear way of how to translate this into the rigid model.

Here we use a slightly different approach. In ref. 49, an analytical expression for the Thiele's force term generated by a single atomic defect that consists of a local modification of anisotropy, **F**_ld_, was found as3
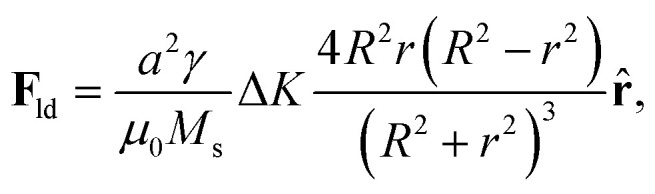
where Δ*K* is the difference between the value of the anisotropy constant at the defect and the one of the plain sample, *K*. *a* is the (squared) lattice constant and *r* is the distance from the position of (the center of) the skyrmion to the defect.

We consider each grain as a surface with an atomic defect density given by *σ*_ld_ = 1/*a*^2^. Each surface differential d*S* generates a differential of force given by *σ*_ld_**F**_ld_d*S*. Then, the force that a grain exerts over a skyrmion is4
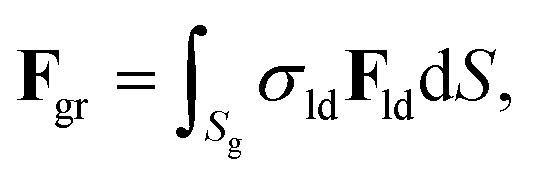
where the integration is done over the grain surface, *S*_g_.

The problem now is that the position and shape of the grains have to be known in order to evaluate this force term. The grain distribution is modeled as follows. Consider that the whole surface of the track (of total surface area 2*WL*) is formed by *N*_g_ grains of square shape and surface area *L*_g_^2^. Thus, *N*_g_ = 2*WL*/*L*_g_^2^. Those grains are randomly distributed over the track with the constraint that there cannot be more than four grain centers in any given square surface of side *L*_g_ (see Fig. 1c).

The typical size of grains is *L*_g_ ≃ 10–20 nm while the radius of skyrmions with the parameters used here (see below) is *R* ≃ 80 nm. Thus, when evaluating the force over a skyrmion due to a given grain its relative orientation with respect to the skyrmion and its shape are not relevant. **F**_gr_ can be thus evaluated as5
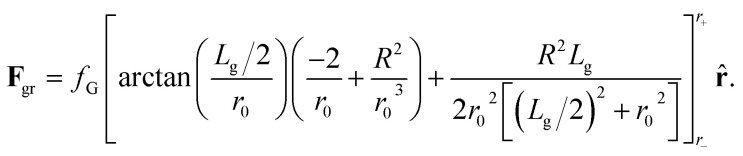


Eqn (5) is found after eqn (4), by arbitrarily choosing that the line segment that goes from the center of the grain to the skyrmion position crosses perpendicularly one of the sides of the grain. We have checked that considering other orientations and shapes the difference in the force is less than 6%. The constant *f*_G_ is *f*_G_ = (2Δ*KγR*^2^)/(*μ*_0_*M*_s_). The variable *r*_0_ should be evaluated at 
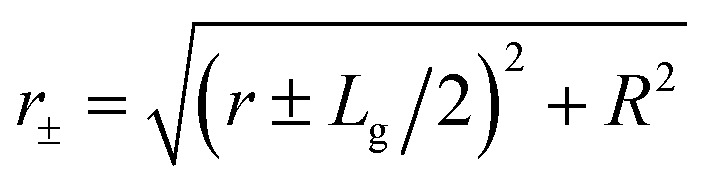
. *r* is now the distance between the grain center and the position of the skyrmion.

Finally, the total force over a skyrmion is the sum of all the grains,6
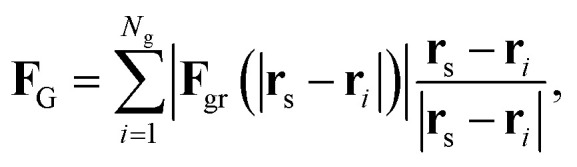
where **r**_s_ is the position of the skyrmion and **r**_*i*_ represents the positions of the centers of the different grains.

To set the value of Δ*K* (needed for evaluating *f*_G_) we follow the same procedure as in the Voronoi tessellation models:^36^ we assume that the distribution of the values of the uniaxial anisotropy constant of the grains follows a normal distribution with mean value *K* and standard deviation *σ*_K_. The value of Δ*K* is given to each grain randomly following the aforementioned normal distribution. Since **F**_G_ is randomly generated, for each set of parameters several simulations with different **F**_G_ are performed and averaged to get statistically significant results.

The granularity model presented yields similar results to those presented, for example, in ref. 27 using a Voronoi tessellation and micromagnetic calculations, justifying the use of Thiele's rigid model approach. However, the key point is that we are able to analytically model the forces due to the grains. This simplifies the numerical treatment of granular systems within the rigid model.

## 3 Fokker–Planck equation

Eqn (1) is a stochastic equation whose dependent variable is the position **r**_s_ of the skyrmion. Its corresponding Fokker–Planck equation can be derived (in a similar way as described in ref. 35):7
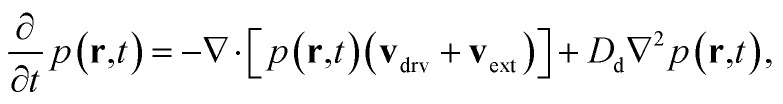
with the definitions8
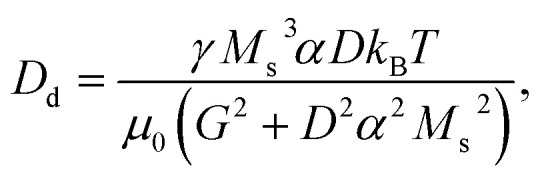
9
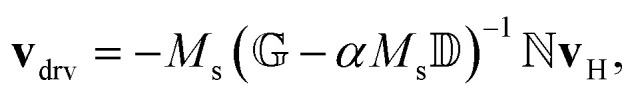
10
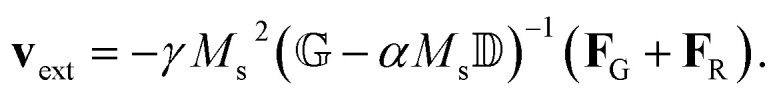


Eqn (7) is a deterministic equation whose solution is *p*(**r**,*t*), the probability density of finding the center-of-mass of the skyrmion at position **r** = (*x*, *y*) at time *t*. It is a convection–diffusion equation. The first term on the right-hand side indicates that the probability density is transported at a velocity **v**_drv_ + **v**_ext_, whereas the second term is a linear, homogeneous, and isotropic diffusion term with constant *D*_d_. Note that each of the solutions of the FPE [eqn (7)] corresponds to infinitely many solutions of the STE [eqn (1)] with the same grain distribution. To achieve the same accuracy as obtained by solving the FPE, the calculation time needed using the STE would be prohibitive. Some recent studies show direct measurements of the probability density cloud.^37^

## 4 Results

Consider a skyrmion, initially at the position (*x*_s_, *y*_s_) = (0, 0), traveling along the racetrack while driven by a **v**_H_ = (0, −*v*_H_). Our purpose is to study how the granularity of the sample, characterized by *L*_g_ and *σ*_K_, affects the skyrmion dynamics at room temperature. To fix numbers, we use **v**_H_ = (0, −426.8) m s^−1^ and *f*_0_ = 8.71 × 10^−14^ m^2^ A^−1^. These parameters are chosen so that, in the clean sample and at *T* = 0 K, the skyrmion travels at about 35 m s^−1^ when far from any border, speeding up to about 100 m s^−1^ when reaching the borders. The probability of surviving along the track at a distance of 1.2 μm is found to be higher than 0.99998. The rest of the parameters used in the simulations are *α* = 0.3, *M*_s_ = 580 kA m^−1^, *K* = 0.425 MJ m^−3^, *R* = 80 nm, *T* = 300 K, *W* = 150 nm, *d* = 0.6 nm, and *L* = 1.2 μm. The radius of the skyrmion is a fixed parameter. One could consider the radius dependence by changing some parameters as a function of radius.^21,50^

**Fig. 2:**
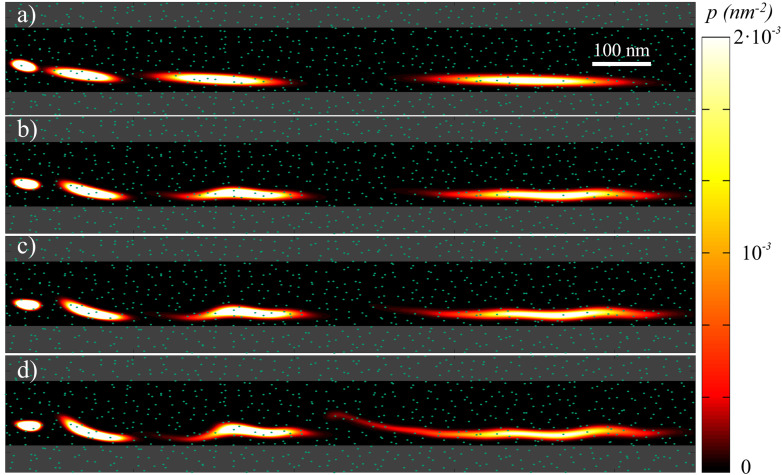
Overlapped snapshots of the probability density *p*(**r**,*t*) for the presence of a skyrmion at a given position **r** along the track for different times (*t* = 1.7, 4.2, 6.7, and 11.7 ns). The distribution of the grains is the same in all figures but *σ*_K_ changes: *σ*_K_ = 0 (a), 0.05*K* (b), 0.1*K* (c), 0.2*K* (d). The side of the grains is *L*_g_ = 20 nm. The dots indicate the grain centers. The gray region indicates where the skyrmion has reached the border, and thus disappeared.

In Fig. 2 we show four simulations considering the same grain distribution, but with different dispersions in the Δ*K* value, from *σ*_K_ = 0 (no granularity) to *σ*_K_ = 0.2*K*. Each plot shows a superposition of four snapshots (at different times) corresponding to the probability density of finding the skyrmion. In this way, the translation and the diffusion effects are clearly visualized. In particular, as *σ*_K_ becomes larger, skyrmions can be trapped or delayed more easily at some grains, stretching the probability density cloud. As a result, the probability of a skyrmion reaching a given distance along the track for a given time is expected to be reduced due to this grain-induced delay effect. In addition, even for infinite times, the probability of a skyrmion reaching a given position is reduced due to the pinning at some grains.

To quantify these results more accurately, the probability that a skyrmion reaches the position *x* of the track before a time *t*, or the skyrmion success probability, is calculated as11
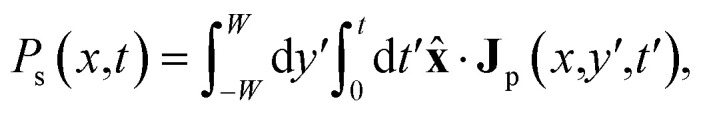
where **J**_p_(*x*,*y*,*t*) is the probability current density. It can be obtained by considering that ∂*p*(**r**,*t*)/∂*t* = −∇**J**_p_. After eqn (7),

12 **J**_p_ = (**v**_drv_ + **v**_ext_)*p*(**r**,*t*) − *D*_d_∇*p*(**r**,*t*).

To have the potential to become a real device, the skyrmionic racetrack should ideally achieve a *P*_s_(*L*,*τ*) = 1 for reasonable times *τ* to be determined by the particular device in mind. This is because in general, one does not desire the skyrmions carrying information to “disappear”, nor to reach their destination beyond the expected time.

**Fig. 3:**
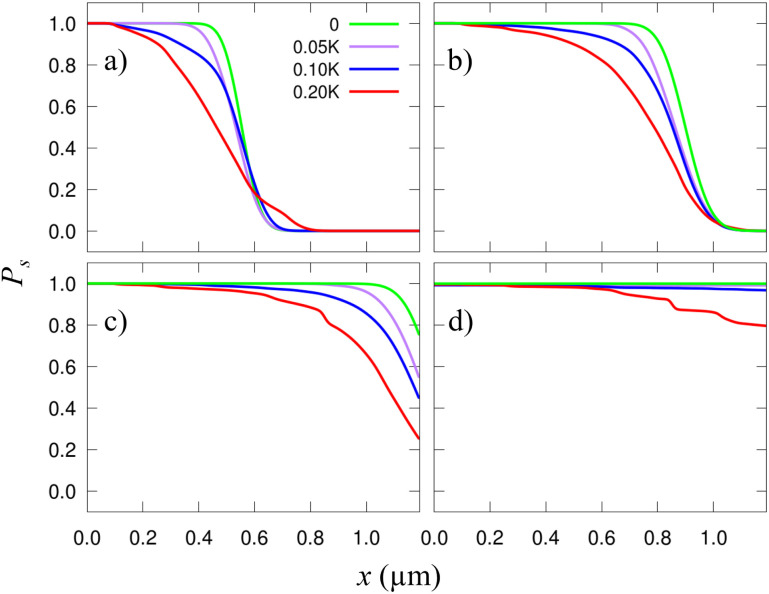
Probability of finding a skyrmion as a function of the traveled length *x* along the racetrack. Each plot corresponds to a different time, from (a) to (d): *t* = 6.67, 10, 13.33, and 20 ns. The different lines correspond to averaging 30 different grain distributions of side *L*_g_ = 20 nm with the same *σ*_K_. *σ*_K_/*K* = 0 (green), 0.05 (purple), 0.1 (blue), and 0.2 (red).

In Fig. 3 we show the skyrmion success probability as it travels along the track for different *σ*_K_. These results have been obtained after averaging the results of 30 simulations [solutions of eqn (7)] considering different distributions of grains with the same size *L*_g_ = 20 nm and *σ*_K_.

**Fig. 4:**
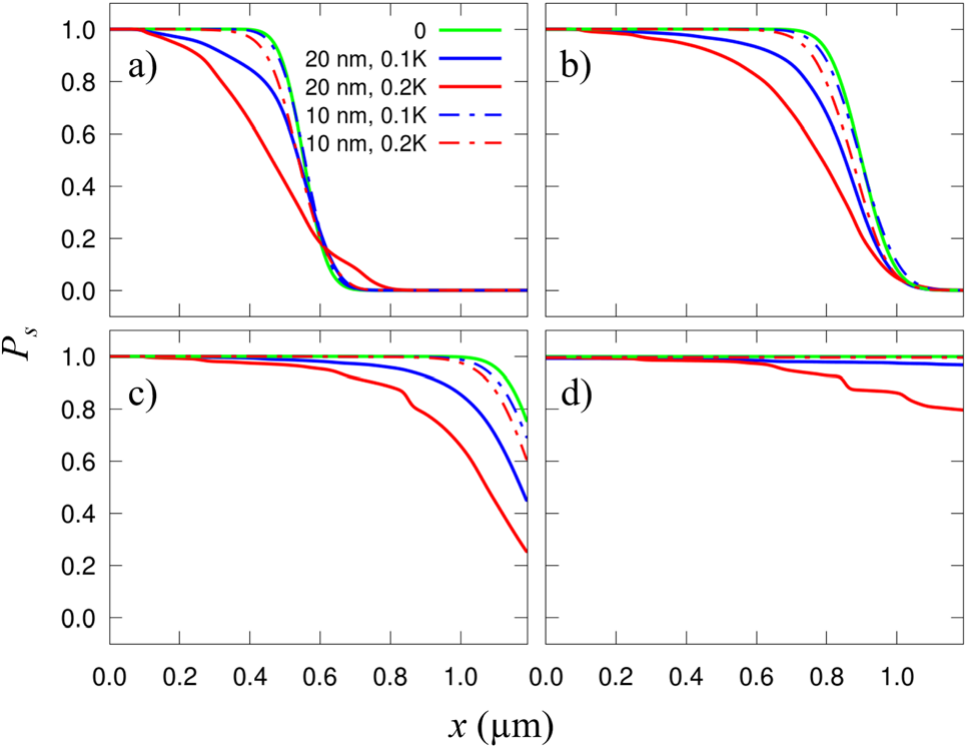
Probability of finding a skyrmion along a racetrack as a function of *x*. Each plot corresponds to a different time, from (a) to (d): *t* = 6.67, 10, 13.33, and 20 ns. Solid blue and red curves are the same as in Fig. 3. The dotted curves of each color correspond to the respective *σ*_K_ but with smaller grains, *L*_g_ = 10 nm. The green solid lines correspond to the clean racetrack and are included for comparison.

In general, finding a skyrmion at a given distance and time is less likely for more heterogeneous samples (larger *σ*_K_) indicating that the granularity somewhat delays the skyrmion. As time goes, the probability of reaching a given distance increases (naturally). After 100 ns (well after the longest time shown in Fig. 3 and 4) we have observed that for the *σ*_K_ = 0.2*K* case (red line in Fig. 3), the probability of reaching the 1.2 μm is about 0.85, indicating that the skyrmion has been lost, pinned, or greatly delayed. It is also worth noting that some of the results found here have also been predicted for antiferromagnetic skyrmions.^43^ The presence of disorder and temperature produces a “hindering” phase, similar to our delay, or to the creep regime described in other studies.

There is a curious effect for small times. As seen in Fig. 3a the distances at which a skyrmion can be found are *larger* for more heterogeneous samples (the red line overcomes the other ones at *x* ≃ 0.6 μm). This is because, when far from the edges, there is a substantial diffusion along the *y* axis and the cloud of probability density stretches significantly along *y*. When the skyrmion reaches the border, it is accelerated along *x*. Thus, the part of the *p*(**r**,*t*) cloud near the edge is then stretched along *x*. As a result, in more heterogeneous samples, there is a relatively large probability of finding skyrmions at larger distances along *x*, with respect to considering a plain sample. At larger times, this effect disappears since the *p*(**r**,*t*) distribution is already mostly concentrated near the borders in all cases.

We also studied how the defect size affects the performance of this device. In Fig. 4 we compare the results already shown in Fig. 3 for *σ*_K_ = 0.1*K* and 0.2*K* with those using a different grain size, *L*_g_ = 10 nm. It is observed that the smaller the grains are, the weaker their effect is. This is because, when the grains are small, the skyrmions are much larger than them, and thus, they seldom perceive the granularity. Actually, from the skyrmion perspective, it moves over an effectively uniform film. This is clearly seen in Fig. 4 where the small-grain results (dashed lines) practically match with the plain sample calculation (green solid line). In this case, if *σ*_K_ were very high, there could be some grain acting basically as a local point defect.^49^ If the grains are much larger than the skyrmions then one would expect that the grain boundaries act as extended defects.^40^

**Fig. 5:**
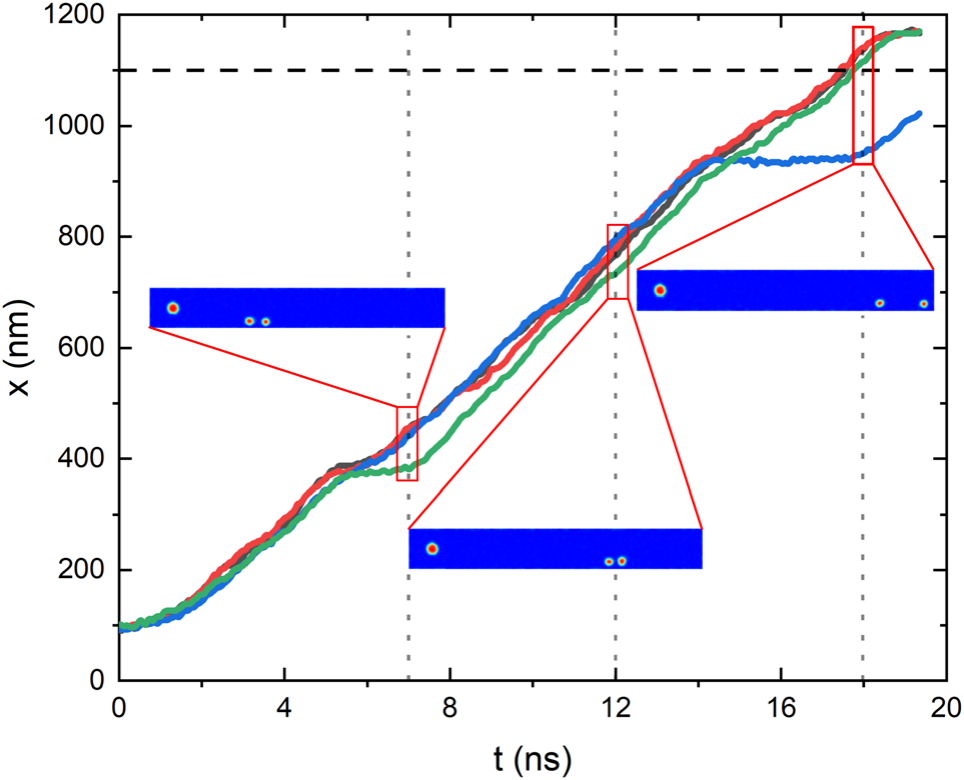
*x*-Position of the center of the skyrmion along the racetrack as a function of time calculated from four (red, green, blue, gray) identical stochastic micromagnetic calculations with the same parameters as discussed in the text. In the insets there are snapshots of the position of different skyrmions at the same time (for the green and blue simulation, together with the initial skyrmion for comparison). The full video for these and other cases can be found in the ESI.†

In order to validate this approach we have performed several micromagnetic calculations using the same geometry and parameters (exchange, saturation magnetization, DM constant, temperature, and *α*) as above and by generating the grains in the track following also the same procedure as described using a position dependent anisotropy constant (see ESI for videos of the simulation results†). The summary of some of these micromagnetic runs can be seen in Fig. 5 where the *x*-position of the center of the skyrmion as a function of time is plotted. The results show that: (i) the skyrmion moves approximately rigidly. Small deformation due to the temperature and the crossing between grains do not destroy the global shape of the skyrmion (although the radius of the skyrmion decreases when approaching the border). We conclude that, while the skyrmion if moving along the track the rigid model is a good approximation with the parameters used. (ii) The results reported in the micromagnetic calculations are consistent with the probabilistic results. In particular, we have found cases in which there is a delay in the movement of the skyrmions due to the granularity, cases where the skyrmions are destroyed at the border, *etc.* The quantitative evaluation of the probability of having these events would be impossible in practice with full micromagnetic approaches, but is just the information that the Fokker–Planck solutions give us.

## 5 Discussion and conclusions

A direct comparison of the presented results with published experimental results is difficult. However, it is worth mentioning that our model predicts a dependence, experimentally found, of the skyrmionic Hall angle with granularity,^15,29^ as seen in Fig. 4 where the skyrmion approaches the border sooner for large granularities. It also predicts the presence of different regimes in the movement of skyrmions: pinning, creep, and flow regimes at low, medium, and large driving currents, respectively.^44^ This is seen in the averaged delay of skyrmions (creep regime) used in the calculations; we also performed simulations at lower driving currents and found that the probability of survival at a given distance went to zero while the probability density plot was fixed around some defects (indicating the pinning regime, not shown). Simulations at larger driving currents (flow regime) also showed a probability of going to zero, but because of the overcoming of the confining edge barriers (not shown).

Here, we have only taken into account anisotropy variations for the grains. In general, other parameters may change from grain to grain (and even the grain boundaries can have an influence on the skyrmion dynamics).^30,36^ To consider all these possibilities the grain modeling could be extended to consider the proper expression in eqn (3) that could be obtained from ref. 18. Nevertheless, from our results, we can conclude that trying to obtain more homogeneous racetracks is more important than obtaining monocrystalline racetracks, in terms of transport efficiency. In this sense, a sample with small grains (relative to the skyrmion size) acts as an effective homogeneous sample.

Of course, stronger variations of anisotropy, softer edge barriers, or other defects could yield skyrmion loss. In particular, Joule heating due to the driving current and the possible temperature gradients created at the joints^44,51,52^ can also affect the dynamics of the skyrmions. We have studied just a few particular cases because our goal was to study the effect of granularity. The main conclusion is that the information transport is not necessarily compromised due to the granularity (except for very heterogeneous samples), although the delay effect could be significant and, thus, precise tracking of skyrmions and time synchronization of the readout would be necessary. For example, in a skyrmionic racetrack memory, a chain of bits is a chain of skyrmions (1) and spaces without skyrmions (0). Hence, preserving the distance between skyrmions is mandatory for the proper functioning of the device. In this sense, racetracks require some kind of “synchronization point” for skyrmions. A possible solution could be considering wedges at the borders.^53^ The overall velocity would be reduced, but feasibility would be ensured.

The methodology presented here can be useful for controlling and somewhat harnessing the randomness in magnetic systems.^54^ The “as realistic as possible” simulations are key ingredients in the design and development of a particular application. Neuromorphic computing systems,^55^ logic and probabilistic computing devices,^56^ or true random number generators^57^ can benefit from the presented results and model.

## Conflicts of interest

There are no conflicts of interest to declare.

## Supplementary Material

NA-005-D3NA00464C-s001

NA-005-D3NA00464C-s002

NA-005-D3NA00464C-s003

NA-005-D3NA00464C-s004

NA-005-D3NA00464C-s005

NA-005-D3NA00464C-s006

NA-005-D3NA00464C-s007

NA-005-D3NA00464C-s008

NA-005-D3NA00464C-s009

NA-005-D3NA00464C-s010

NA-005-D3NA00464C-s011

NA-005-D3NA00464C-s012

NA-005-D3NA00464C-s013
