# Supplementary figures and images for: Survival of skyrmions along granular racetracks at room temperature
Source: Nanoscale Adv. 2023 Jul 28;5(18):4728–34. doi: 10.1039/d3na00464c (PMC10496888; doi:10.1039/d3na00464c)

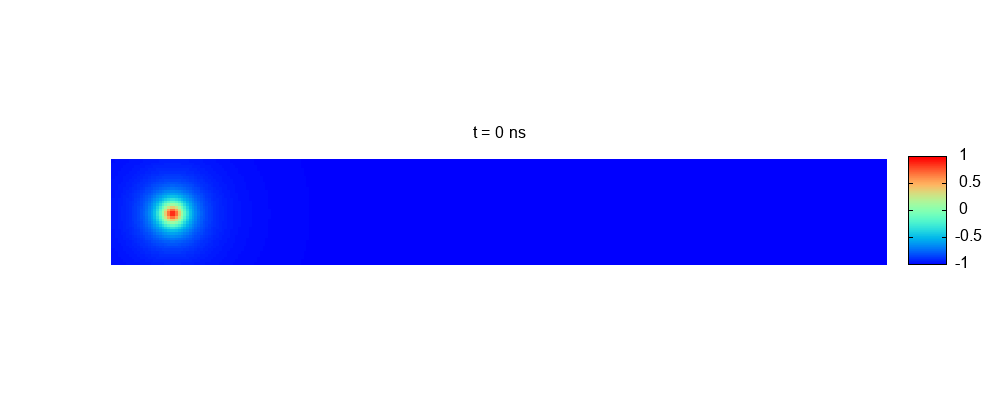

Supplement: NA-005-D3NA00464C-s001 [file NA-005-D3NA00464C-s001.gif]

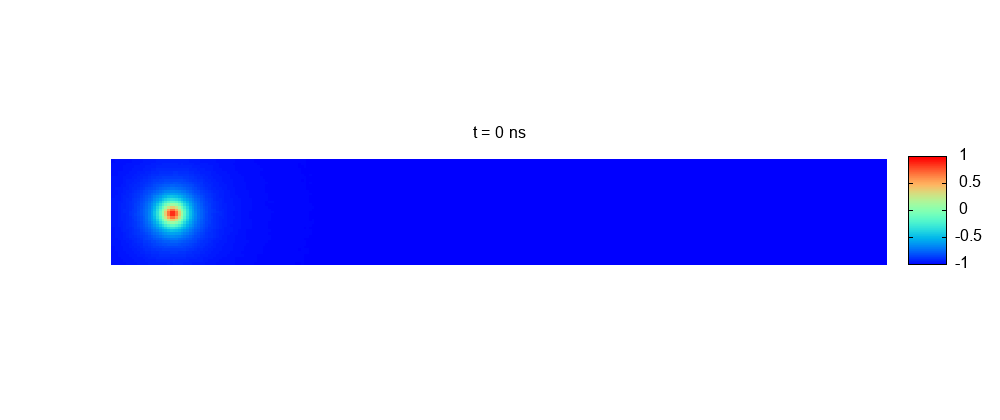

Supplement: NA-005-D3NA00464C-s002 [file NA-005-D3NA00464C-s002.gif]

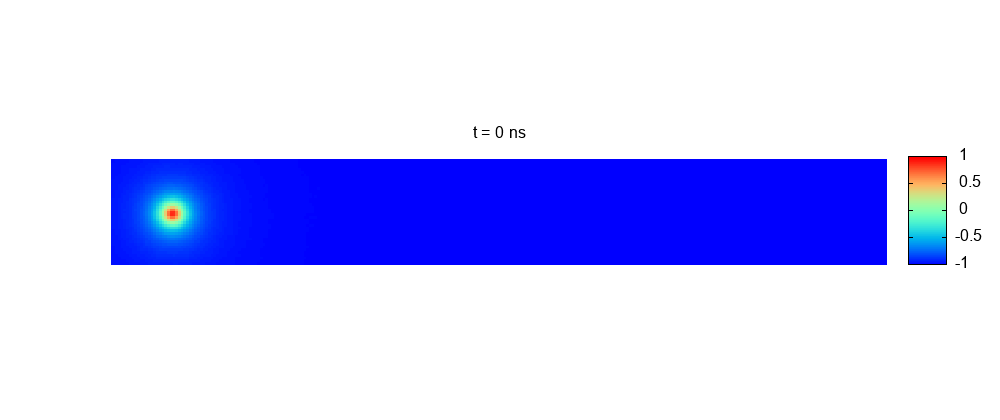

Supplement: NA-005-D3NA00464C-s003 [file NA-005-D3NA00464C-s003.gif]

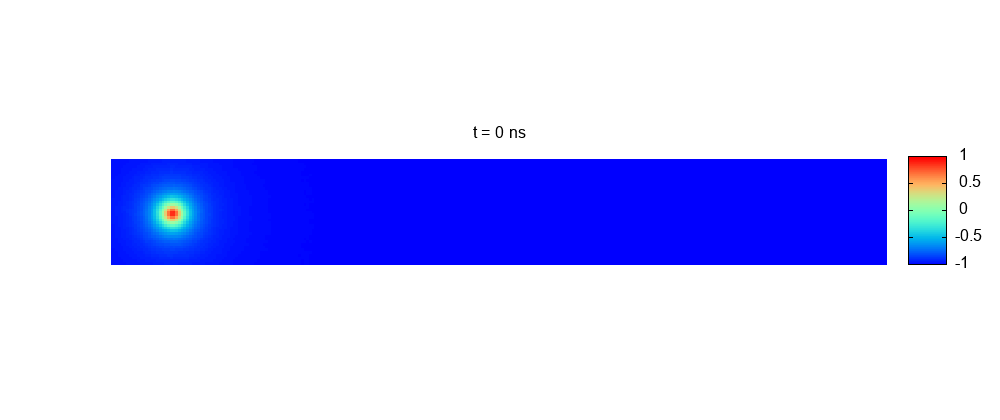

Supplement: NA-005-D3NA00464C-s004 [file NA-005-D3NA00464C-s004.gif]

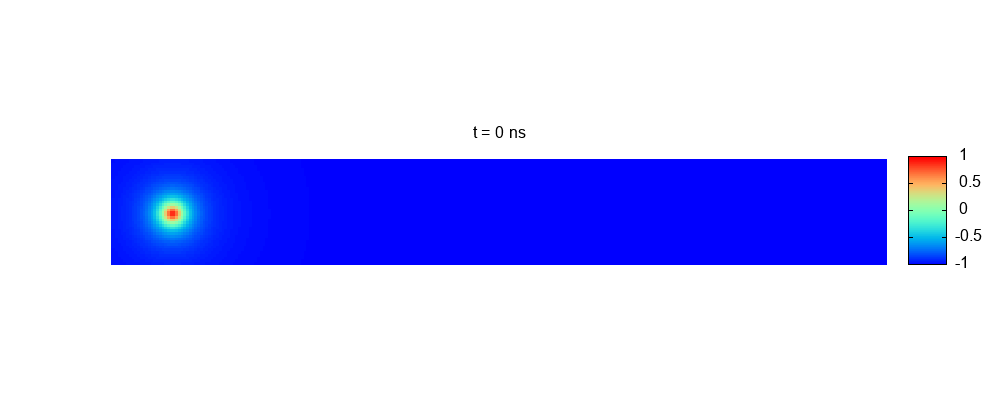

Supplement: NA-005-D3NA00464C-s006 [file NA-005-D3NA00464C-s006.gif]

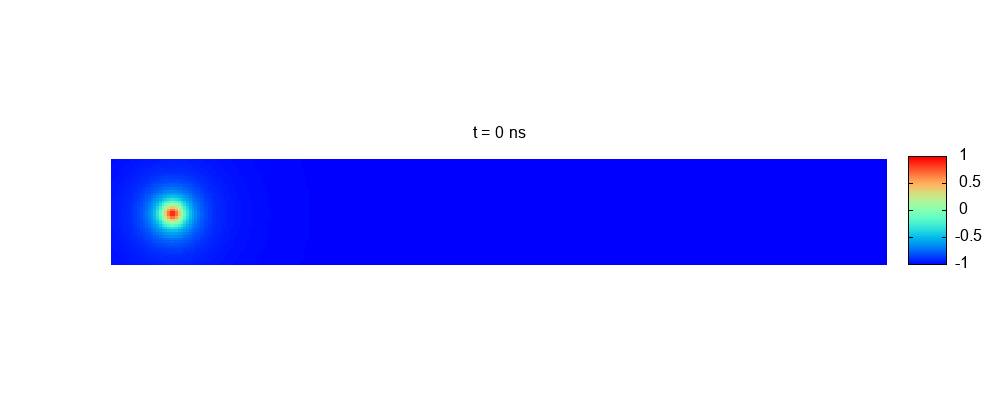

Supplement: NA-005-D3NA00464C-s007 [file NA-005-D3NA00464C-s007.gif]

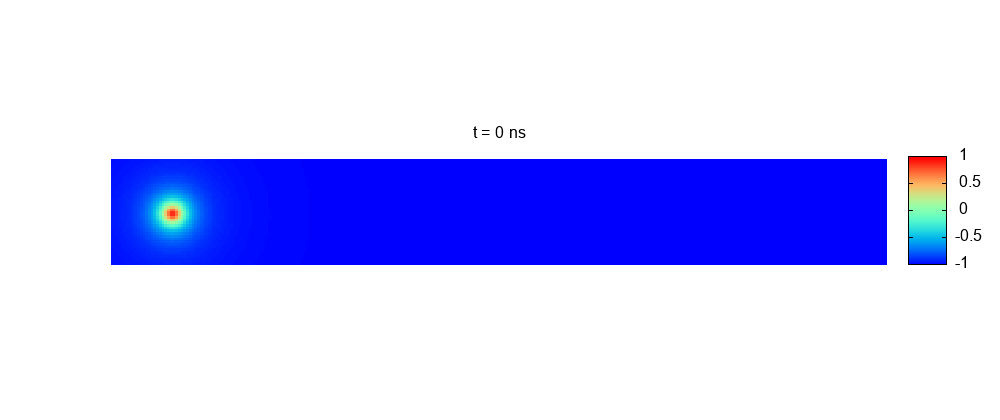

Supplement: NA-005-D3NA00464C-s008 [file NA-005-D3NA00464C-s008.gif]

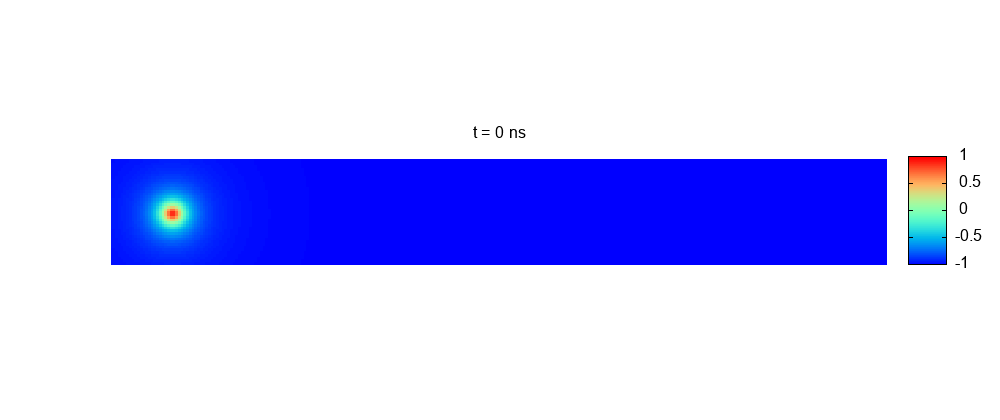

Supplement: NA-005-D3NA00464C-s009 [file NA-005-D3NA00464C-s009.gif]

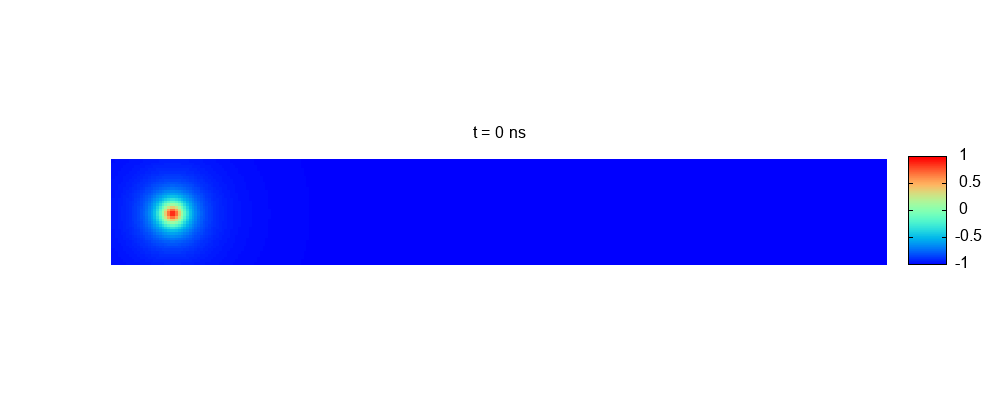

Supplement: NA-005-D3NA00464C-s010 [file NA-005-D3NA00464C-s010.gif]

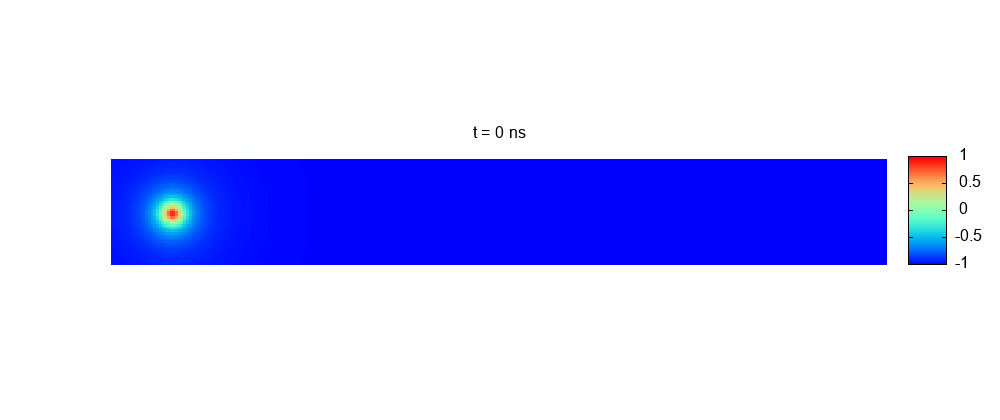

Supplement: NA-005-D3NA00464C-s012 [file NA-005-D3NA00464C-s012.gif]

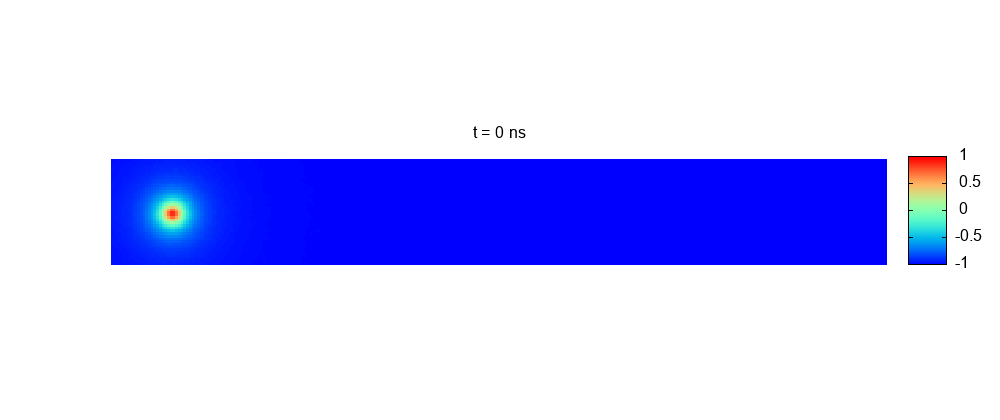

Supplement: NA-005-D3NA00464C-s013 [file NA-005-D3NA00464C-s013.gif]
