# Supplementary material for: Survival of skyrmions along granular racetracks at room temperature
Source: Nanoscale Adv. 2023 Jul 28;5(18):4728–34. doi: 10.1039/d3na00464c (PMC10496888; doi:10.1039/d3na00464c)
Supplement: NA-005-D3NA00464C-s005 [file NA-005-D3NA00464C-s005.pdf]

Electronic Supplementary Material (ESI) for Nanoscale Advances.
This journal is © The Royal Society of Chemistry 2023

Supplementary material for

"Survival of skyrmions along granular racetracks at room temperature"

Josep Castell-Queralt, Guillermo Abad-López, Leonardo González-Gómez, Nuria Del-Valle, and Carles Navau

To validate Thiele's approximation, we have performed several simulations using the stochastic Landau-Lifshitz-Gilbert equations. There are two sets of simulations (slow and fast), each of them done with exactly the same parameters. There is no difference in the parameters and the difference in the results comes from the stochasticity of the movement.

On of the sets of results (slow) are performed using the same driving current densitiy as in the main text. In this case the probability that the skyrmions is destroyed at the border is quite small as predicted within the Fokker-Planck (FP) formalism. In the simulations done we did not find skyrmion annihilation at borders. Moreover, the skyrmion can be partially pinned at different grains, delaying the skyrmions, also as predicted within the FP model, as explained in the main text. See the videos "slow_[color].gif".

We have also performed simulations with double driven current density, showing that in these cases, in some simulations the skyrmions is annihilated at the borders, whereas in some others it is not. See the videos "fast_[color].gif".

We stress that even though the extreme conditions (room temperature, granularity and high driving-current densities) the skyrmion somehow maintains its shape justifying the use of Thiele's equation.
